# Irregular Expression of Cellular Stress Response Markers in the Placenta of Women with Chronic Venous Disease

**DOI:** 10.3390/antiox11112277

**Published:** 2022-11-17

**Authors:** Cielo García-Montero, Oscar Fraile-Martinez, Sonia Rodriguez-Martín, Rosa M. Funes Moñux, Jose V. Saz, Coral Bravo, Juan A. De Leon-Luis, María Ruiz-Minaya, Leonel Pekarek, Miguel A. Saez, Alberto García-Lledo, Melchor Alvarez-Mon, Julia Bujan, Natalio García-Honduvilla, Miguel A. Ortega

**Affiliations:** 1 Department of Medicine and Medical Specialities, Faculty of Medicine and Health Sciences, University of Alcalá, 28801 Alcala de Henares, Spain; 2 Ramón y Cajal Institute of Sanitary Research (IRYCIS), 28034 Madrid, Spain; 3 Service of Pediatric, Hospital Universitario Principe de Asturias, 28801 Alcala de Henares, Spain; 4 Department of Biomedicine and Biotechnology, Faculty of Medicine and Health Sciences, University of Alcalá, 28801 Alcala de Henares, Spain; 5 Department of Public and Maternal and Child Health, School of Medicine, Complutense University of Madrid, 28040 Madrid, Spain; 6 Department of Obstetrics and Gynecology, University Hospital Gregorio Marañón, 28009 Madrid, Spain; 7 Health Research Institute Gregorio Marañón, 28009 Madrid, Spain; 8 Pathological Anatomy Service, Central University Hospital of Defence—UAH Madrid, 28801 Alcala de Henares, Spain; 9 Cardiology Service, University Hospital Príncipe de Asturias, 28806 Alcala de Henares, Spain; 10 Immune System Diseases-Rheumatology and Internal Medicine Service, University Hospital Príncipe de Asturias, CIBEREHD, 28806 Alcala de Henares, Spain

**Keywords:** chronic venous disease, placenta, oxidative stress, NRF2, KEAP1, CUL3, GSK-3β

## Abstract

Pregnancy comprises a period in a woman’s life in which the circulatory system is subjected to hemodynamical and biochemical changes. During this period, while restructuring blood vessels and exchanging maternal-fetal products there is an increased risk of developing chronic venous disease (CVD), which may have an echo in life after childbirth for both mother and child. Previously, we investigated that pregnancy-associated CVD involves changes in placental architecture at angiogenesis, lymphangiogenesis and villi morphology compared with healthy controls (HC) with no history of CVD. We aimed to more deeply investigate the oxidative stress response in placenta from women with CVD versus HC through several markers (NRF2, KEAP1, CUL3, GSK-3β). An observational, analytical, and prospective cohort study was conducted on 114 women in their third trimester of pregnancy (32 weeks). A total of 62 participants were clinically diagnosed with CVD. In parallel, 52 controls with no history of CVD (HC) were studied. Gene and protein expressions of NRF2, KEAP1, CUL3, GSK-3β were analyzed by real-time polymerase chain reaction (RT-qPCR) and immunohistochemistry. Nrf2 gene and protein expression was significantly greater in placental villi of women with CVD, while Keap1, CUL-3 and GSK-3β gene and protein expressions were significantly lower. Our results defined an aberrant gene and protein expression of Nrf2 and some of their main regulators Keap1, CUL-3 and GSK-3 β in the placenta of women with CVD, which could be an indicator of an oxidative environment observed in this tissue.

## 1. Introduction

Pregnancy comprises a period in a woman’s life in which the circulatory system is subjected to hemodynamical, mechanical and biochemical changes [1,2,3,4]. During this period, while restructuring blood vessels and exchanging maternal-fetal products, there is an increased risk of developing chronic venous disease (CVD) [5,6]. This can be due to the fact that the diameter of veins tends to increase throughout pregnancy [7]. However, notwithstanding a significant decrease can be observed after childbirth, veins do not return to their initial diameter, particularly in women undergoing CVD during pregnancy [8]. Approximately, 40% of pregnant women suffer from CVD and the risk of suffering from this condition can increase according to the number of pregnancies [9]. During the course of CVD, venous return from lower extremities becomes difficult, as there is an ambulatory venous hypertension progressing over time [10,11,12]. CVD can be classified according to the Clinical-Etiology-Anatomy-Pathophysiology (CEAP) criteria based on the clinical manifestations [13]. The most prevalent clinical groups of CVD are C0 (no clinical evidence): 9%, C1 (telangiectasias, reticular veins): 26%, and C2 (varicose veins -VVs-): 19%. Some relevant findings in this sense were the highest prevalence of C2 in Western Europe, with a growing incidence and seriousness of this chronic disease [14,15].

Previous studies have noticed that CVD supposes a systemic stress during pregnancy that impairs placenta structure and function. For instance, it has been observed that pregnancy-associated CVD is associated with changes at placental villous architecture and enhanced apoptotic death [16]. Likewise, pregnancy-associated CVD involves changes at angiogenesis, lymphangiogenesis, with several changes in the extracellular matrix compared with healthy controls (HC) with no history of CVD [17,18]. All in all, these factors could have a negative impact not only on the mother’s life—during and after childbirth—but also on the fetuses’ development [19].

In this sense, the nuclear factor erythroid 2-related factor 2 (NRF2 or *NFE2L2*) is a critical transcription factor that is involved in cellular stress response, modulating the expression of several gene products [20]. Likewise, it appears to be a major modulator of redox homeostasis and permits cell adaptation under oxidative stress [21]. Importantly, augmented detection of various oxidative stress markers in maternofetal structures and in the serum of women with CVD has also been previously reported [22,23]. Furthermore, KEAP1 (kelch such as ECH associated protein 1), CUL3 (cullin 3) and GSK-3β (glycogen synthase kinase 3 beta) are major regulators of Nrf2 function and activation [24,25]. The role of Nrf2 and their main regulators has been well-established in the pathogenesis of vascular and obstetric diseases [26,27].

Thus, the purpose of this research work is to pursue potential alterations in these stress-related markers (Nrf2, Keap1, Cul3, GSK3-β) in placenta from women with CVD and compare them with placenta from HC for a deeper understanding of the pathophysiological network in CVD and its possible implications, especially in pregnancy.

## 2. Material and Methods

### 2.1. Experimental Design

In the present work, an observational, analytical, prospective study was carried out with 114 women in the last trimester of pregnancy. Of them, 62 with a clinical diagnosis of CVD according to the CEAP classification [13] and 52 women with no history of CVD (referred as HC) were included. The median age in the CVD group was 33 (with an interquartile range (IQR) of 22–40 years) whereas HC presented a median age of 34 (IQR, 27–41 years). The median gestational period for CVD women was 40.5 weeks (with an IQR of 39–41.5 weeks) and 41 weeks for HC (IQR, 39–42 weeks).

Prior to enrolment, each patient was properly informed, providing signed written consent. This work passed the Clinical Research Ethics Committee of the Central University Hospital of Defense University of Alcalá (37/17) and was performed according to the ethical principles of autonomy, beneficence, non-maleficence and distributive justice, as well as following the regulations of Good Clinical Practice, the principles of the Declaration of Helsinki (2013) and the Oviedo Convention (1997).

Women included in our study were >18 years old, with medical presentation of CVD (CEAP ≥ 1) in their lower limbs during the third trimester. Exclusion criteria included women with previous evidence of (1) arterial hypertension; (2) venous malformations; (3) heart, kidney or lung failure; (4) BMI ≥ 25; (5) clinical diagnosis of type 1/type 2/gestational diabetes mellitus or other endocrine diseases; (6) autoimmune diseases; (7) active infectious processes; (8) habitual alcohol (≥1 unit/day), tobacco (≥1 cigarette/day), or drug consumption; (9) preeclampsia and/or HELLP syndrome; (10) fetal growth restriction of known causes; (11) Presence of placental infarction, avascular villi, delayed villus maturation, or chronic villitis; (12) Evidence of CVD before pregnancy and (13) the appearance of any exclusion criteria until delivery.

### 2.2. Clinical Evaluations

In third trimester consultation, clinical history for each woman was revised and physical examinations were accomplished. Lower limbs ultrasounds were evaluated with Eco-Doppler (Portable M-Turbo Eco-Doppler; SonoSite, Inc., Bothell, WA, USA) at 7.5 MHz.

With respect to the sociodemographic characteristics of the sample studied, there were no significant differences between CVD and their HC regarding the number of pregnancies: 33 (53.2%) in the event of women with CVD and 19 (36.5%) for the HC group (Table 1). Furthermore, both groups did not present any statistical differences in their clinical features (gestational age, c-section delivery, prior pregnancies and abortions, regularity of menstrual cycles and sedentary profession (Table 1).

### 2.3. Tissue Samples

Postpartum placental biopsies were collected from all 114 patients. For each patient sample, five placental pieces were obtained using a scalpel to include diverse mixed cotyledons and then they were separated in two different sterile tubes: one containing minimal essential medium (MEM; Thermo Fisher Scientific, Inc., Waltham, MA, USA) with 1% antibiotic/antimycotic (streptomycin, amphotericin B, and penicillin; Thermo Fisher Scientific, Inc.) and another with RNAlater^®^ solution (Ambion; Thermo Fisher Scientific, Inc., Waltham, MA, USA). Thereafter, the samples were processed in a class II laminar flow hood (Telstar AV 30/70 Müller 220 V 50 MHz; Telstar; Azbil Corporation) in a sterile environment. Afterwards, samples were preserved in 1 mL of RNAlater^®^ at −80 °C for later processing for gene expression study.

Then, conserved MEM samples were washed and rehydrated five times in MEM free of antibiotics to eliminate erythrocytes. Then, by using a scalpel they were divided into 2 cm fragments and fixed in F13 (60% ethanol, 20% methanol, 7% polyethylene glycol and 13% distilled water), according to collected protocols [18]. Subsequently, paraffin-embedded samples were formed using molds. After the paraffin was solidified, an HM 350 S rotary microtome (Thermo Fisher Scientific, Inc., Waltham, MA, USA) was employed to obtain 5 µm thick sections. Thereafter, they were stretched in a hot water bath and collected on glass slides treated with 10% polylysine, facilitating the adherence of the sections.

### 2.4. Gene Expression Studies Using Reverse Transcription-Quantitative PCR (RT-qPCR)

First, we extracted RNA through the guanidinium thiocyanate-phenol-chloroform method as described in previous studies [28]. This method allows the study of the mRNA expression levels of selected genes.

Complementary DNA (cDNA) was synthesized by reverse transcription (RT) from 50 ng/µL of RNA samples. A total of 4 µL of each sample was mixed with 4 µL of 0.25 µg/µL oligo-dT solution (Thermo Fisher Scientific, Inc., Waltham, MA, USA) and afterwards placed at 65 °C for 10 min in a dry bath (AccuBlock, Labnet International Inc., Edison, NJ, USA), leading to the denaturation of RNA. The samples were then placed on ice and 10 µL of reverse transcription mix containing the following products: 2.8 µL of First Strand Buffer 5X (250 mM Tris-HCl and pH 8.3; 375 mM KCl:15 mM MgCl_2_) (Thermo Fisher Scientific, Inc., Waltham, MA, USA); 1 µL RT enzyme (all from Thermo Fisher Scientific, Inc., Waltham, MA, USA); 2 µL of 10 mM deoxyribonucleotide triphosphate; 2 µL of 0.1 M dithiothreitol; 1.7 µL of DNase and RNase free water; 0.5 µL of RNase inhibitor (RNase Out).

The reverse transcription process was performed with a G-Storm GS1 thermocycler (G-Storm Ltd., Middlesbrough, UK). The samples were then incubated at 37 °C for one hour and fifteen min in order to facilitate cDNA synthesis. At this point, the temperature was increased to 70 °C and held for 15 min, causing reverse transcriptase denaturation. Subsequently, the temperature was gradually lowered to 4 °C. Negative reverse transcription was equally carried out to ensure the absence of genomic DNA contamination in RNA samples in which the M-MLV RT enzyme was substituted by DNase- and RNase-free water. cDNA produced at room temperature was diluted 1:20 in DNase- and RNase-free water and stored at −20 °C until further use.

Specific primers for the selected genes (Table 2) were designed de novo through the Primer-BLAST and AutoDimer online applications [29,30]. TATA-box binding protein (TBP) gene, which is constitutively expressed, was used as a control to normalize the results [31]. The gene expression units are expressed as relative quantities of mRNA. RT-qPCR was performed on a StepOnePlus™ System (Applied Biosystems; Thermo Fisher Scientific, Inc.) using the relative standard curve method. The reaction was completed as follows: 5 µL of sample—mixed at 1:20 with 10 µL iQ™ SYBR^®^ Green Supermix (Bio-Rad Laboratories, Inc Hercules, California, USA.)—was mixed with 1 µL each forward and reverse primers, and 3 µL of DNase and RNase-free water, which were then added to a MicroAmp^®^ 96-well plate (Applied Biosystems; Thermo Fisher Scientific, Inc., Waltham, MA, USA). Thermocycling conditions were used: Initial denaturation for 10 min at 95 °C, denaturation for 15 s at 95 °C, annealing at variable temperatures depending on the melting temperature of each primer pair for 30 s, and elongation at 72 °C for 1 min, for 40–45 cycles. Then, a dissociation curve for 15 s at 95 °C, 1 min 60 °C, 15 s 95 °C, and 15 s 60 °C was developed. Fluorescence detection was performed at the end of every repeat cycle (amplification) and at the different steps of the dissociation curve. The data collected from the aforementioned genes were included in a standard curve made by serial dilutions of a mixture of the samples, that were included in each plate according to the constitutive expression of TBP (in agreement with the manufacturer’s protocols). This RT-qPCR was performed twice in all samples of placenta tissue.

### 2.5. Immunohistochemical Analysis

Avidin-biotin complex with avidin peroxidase was used for antigen/antibody reactions detection [17]. Immunohistochemical studies were performed on placenta samples embedded in paraffin. The antibodies used are detailed in the protocol specifications (Table 3).

Placental tissues were incubated with the primary antibody for one hour and a half (Table 3). Subsequently, it was incubated with 3% BSA Blocker (cat. no. 37,525; Thermo Fisher Scientific, Inc., Waltham, MA, USA) and PBS (4 °C overnight). The next day, placental samples were incubated with biotin-conjugated secondary antibody, previously diluted in PBS during one hour and a half at room temperature (Table 3). Thereafter, the avidin-peroxidase conjugate ExtrAvidin^®^-Peroxidase (Sigma-Aldrich; Merck KGaA, San Luis, MO, USA) was added for one hour at room temperature (1:200 dilution in PBS). Finally, chromogenic diaminobenzidine (DAB) substrate kit (cat. no. SK-4100; Maravai LifeSciences, San Diego, CA, USA), was used to determine protein expression level. This kit was prepared immediately before exposure (5 mL distilled water; four drops DAB; two drops of hydrogen peroxide and two drops of buffer).

The use of the peroxidase chromogenic substrate for 15 min at room temperature allowed for the detection of the signal in form of brown stains. For each section, negative controls were assigned for the different proteins, replacing incubation with primary antibody for a PBS solution. Carazzi hematoxylin was used for 15 min to achieve contrast in all tissues.

### 2.6. Microscopic Observation and Statistical Analysis

A total of 5 different sections and 10 areas of view were randomly examined for every patient of CVD and HC groups. A patient was reported as positive when the stained mean area in the studied sample was ≥5% of the total, following the immunoreactive score (IRS) as established in previous studies [22]. Immunostaining was evaluated by two independent histologists, and then each sample was scored using the following scale: 0–1, minimum staining (≤25%); 2, moderate staining (25–65%); and 3–4, strong staining (≥65–100%). A Zeiss Axiophot optical microscope (Carl Zeiss, Oberkochen, Germany) was used for the histological determination.

For the treatment of statistical data, GraphPad Prism^®^ v6.0 (GraphPad, Inc., San Diego, CA, USA) program was utilized. Mann–Whitney U test allow the comparison between both groups, being expressed as the median ± SEM. Significant values were established as *p* < 0.05 (*), *p* < 0.01 (**), and *p* < 0.001 (***).

## 3. Results

### 3.1. The Placental Villi of Pregnant Women with CVD Display an Overexpression in the NRF2 Marker

**Figure 1:**
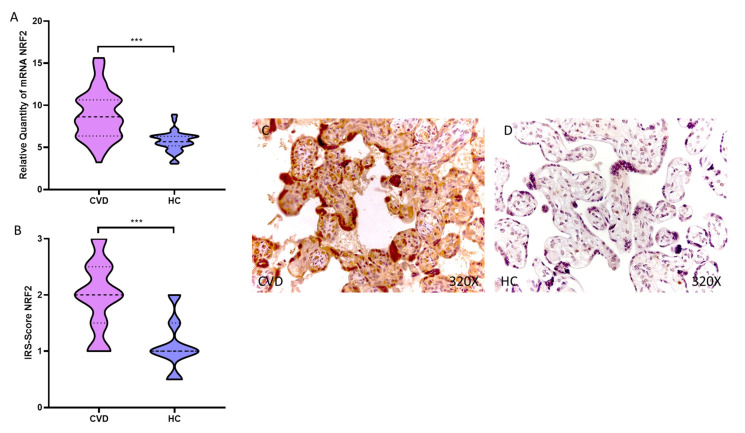
(**A**). NRF2 mRNA expression in the CVD patients (chronic venous disease) and HC group (Healthy controls). (**B**). IRS-Scores for NRF2 in the placenta villi of the CVD group and the HV group. (**C**,**D**). Images showing the immunostaining for NRF2 in the placenta villi of the CVD group and the HV group. *p* < 0.001 (***).

Our results show a statistically significative augmentation in NRF2 gene expression in the placental tissue of women undergoing CVD during pregnancy, *** *p* < 0.0001 [CVD = 8.863 ± 2.893, HC = 5.734 ± 1.168, Figure 1A]. Histological analysis of the placental villi showed that the placental villi of women with CVD showed a significant increase in protein expression of NRF2, *** *p* < 0.0001 [CVD = 1.976 ± 0.617, HC = 1.115 ± 0.450, Figure 1B]. Protein expression of NRF2 was strongly displayed throughout the placental villi of women affected by CVD in comparison to HC (Figure 1C,D).

### 3.2. KEAP1, CUL3 and GSK-3β Expression Level Is Increased in the Placental Villi of Women with CVD during Pregnancy

**Figure 2:**
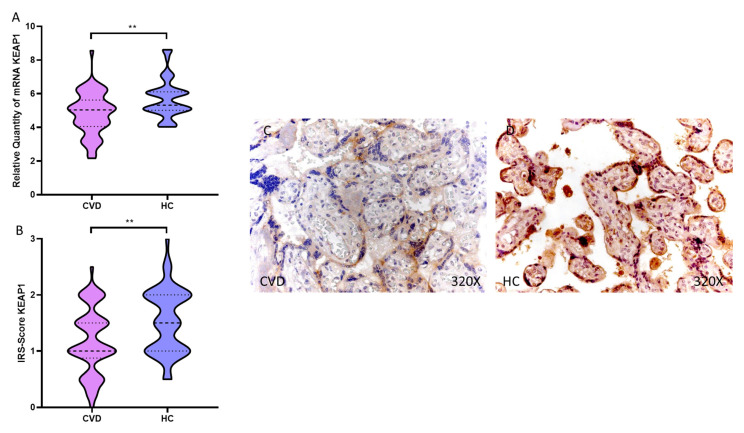
(**A**). KEAP1 mRNA expression in the CVD patients (chronic venous disease) and HC group (Healthy controls). (**B**). IRS-Scores for KEAP1 in the placenta villi of the CVD group and the HV group. (**C**,**D**). Images showing the immunostaining for KEAP1 in the placenta villi of the CVD group and the HV group. *p* < 0.01 (**).

We observed how the placental villi of women with CVD showed a significant decrease in KEAP1 gene expression compared to HC, ** *p* = 0.0015 [CVD = 4.816 ± 1.238, HC = 5.728 ± 1.158, Figure 2A]. This significant decrease was maintained when performing the histological analysis of the placental villi of women with CVD, ** *p* = 0.0021 [CVD = 1.173 ± 0.583, HC = 1.538 ± 0.576, Figure 2B]. Protein expression of KEAP1 was strongly displayed in the entire placental villus of female HC compared to CVD (Figure 2C,D).

**Figure 3:**
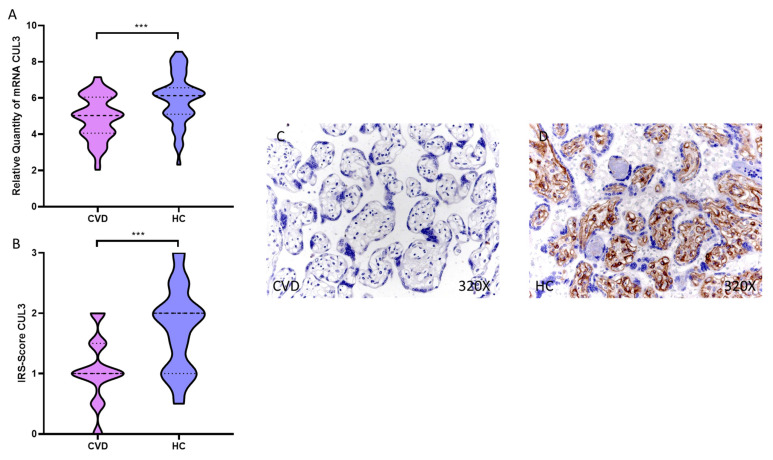
(**A**). CUL3 mRNA expression in the CVD patients (chronic venous disease) and HC group (Healthy controls). (**B**). IRS-Scores for CUL3 in the placenta villi of the CVD group and the HV group. (**C**,**D**). Images showing the immunostaining for CUL3 in the placenta villi of the CVD group and the HV group. *p* < 0.001 (***).

Our results show a significant decrease in CUL3 gene expression in the placental villi of women with CVD compared to HC, *** *p* < 0.0001 [CVD = 4.904 ± 1.181, HC = 5.952 ± 1.376, Figure 3A]. Histological analysis of the placental villi showed how the placental villi of women with CVD showed a significant decrease in protein expression of CUL3, *** *p* < 0.0001 [CVD = 1.060 ± 0.533, HC = 1.740 ± 0.686, Figure 3B]. Protein expression of CUL3 was shown to be intense in the extracellular matrix and around the fetal capillaries of the placental villus of HC compared to CVD women (Figure 3C,D).

**Figure 4:**
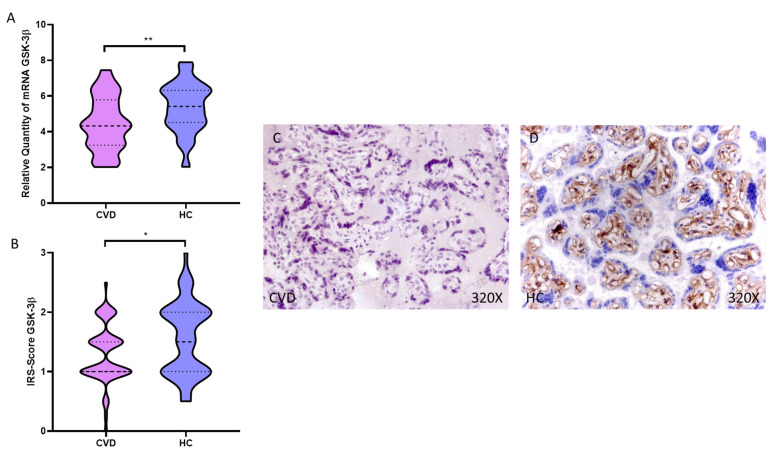
(**A**). GSK-3β mRNA expression in the CVD patients (chronic venous disease) and HC group (Healthy controls). (**B**). IRS-Scores for GSK-3β in the placenta villi of the CVD group and the HV group. (**C**,**D**). Images showing the immunostaining for GSK-3β in the placenta villi of the CVD group and the HV group. *p* < 0.05 (*), *p* < 0.01 (**).

In this sense, we have observed how the placental villi of women with CVD showed a significant decrease in the gene expression of GSK-3β compared to HC, ** *p* = 0.0015 [CVD = 4.816 ± 1.238, HC = 5.728 ± 1.158, Figure 4A]. This significant decrease was maintained when performing the histological analysis of the placental villi of women with CVD, ** *p* = 0.0021 [CVD = 1.173 ± 0.583, HC = 1.538 ± 0.576, Figure 4B]. Protein expression of GSK-3β was intensely displayed in the extracellular matrix and around the fetal capillaries of the placental villus of HC compared to CVD women (Figure 4C,D).

## 4. Discussion

Our results indicate an aberrant expression of the transcription factor Nrf2 and its main regulators CUL-3, Keap1 and GSK-3β in the placenta tissue of women with CVD. This study gains further insights into the pathophysiological changes occurred in this organ during pregnancy, supporting the relevance of suffering from CVD as a possible obstetric complication for maternofetal wellbeing, as suggested by previous studies [32,33].

Firstly, we detected an enhanced Nrf2 expression in the placenta of women with CVD in comparison with their healthy controls. Nrf2 is a major transcription factor which regulates the expression of several genes involved in the cellular stress response. Specifically, Nrf2 participates in the “phase 2 response”, also known as “the electrophile counterattack response”, modulating the expression of some “vitagenes”, such as heme oxygenase 1, thioredoxin or thioredoxin reductase, and counteracting the damaging effects of oxidants and electrophiles [34]. Thus, Nrf2 is critical for a proper antioxidant response in the organism, also influencing other biological processes, such as detoxification, metabolism and inflammation [35,36,37]. Because of that, it is widely accepted that disturbances in the Nrf2 system represent a major driver of several pathologies, including inflammatory, metabolic and cardiovascular disorders [38]. Likewise, Nrf2 overexpression or downregulation has been observed in several adverse outcomes of pregnancy, including gestational diabetes mellitus, intrauterine growth restriction, reproductive toxicity, preeclampsia, and preterm birth, having detrimental consequences on the trophoblast´s behavior and function [27]. In our work, oxidative stress is a major feature of many obstetric complications, driving cellular damage of nucleic acids, lipids and proteins [39,40,41]. This could have potential pathogenic implications not only for the mother but also for the fetus, driving significant changes in fetal programming via epigenetic mechanisms [42]. Conversely, prior studies have shown that reduced activity of Nrf2 seems to increase angiogenesis in placental tissue and improved maternal and fetal outcomes in animal models of preeclampsia, supporting the dual role that oxidative stress may play in pregnancy complications [43]. Sources of oxidative stress can be different according to the obstetric complication. For instance, in the event of preeclampsia, the placenta represents the main producers of free radicals and oxidative stress [44]. As previously reported, patients with CVD often show evidence of local and systemic oxidative stress [45], and this appears to have detrimental effects on the placenta and umbilical cord [22,23], along with an enhanced detection of lipid peroxidation in the maternal blood and a decrease of fetal pH [23]. Considering these results, we propose that CVD is associated with systemic oxidative stress, possibly entailing detrimental consequences for maternofetal wellbeing. Thus, Nrf2 may be an indicator of the exposure to significant stressors in the placenta tissue related to CVD, although consequences of Nrf2 dysregulation in pregnancy complications remain to be fully understood [27].

There are several products that can regulate Nrf2 activation and function. For instance, the PI3K/Akt/mTOR pathway appears to be a major activator of basal and induced Nrf2 activities [46]. An enhanced expression of this pathway has been previously reported in the placental tissue of women with CVD [47], aiding to explain the increased expression of Nrf2. Likewise, Notch signaling also appears to upregulate Nrf2 expression [48]. Despite the canonical Notch pathway havingnot been studied in the placenta of women with CVD, the non-canonical pathway is responsible for the activation of Wnt/β-catenin signaling, which appears to be upregulated in these patients [47,49].

Conversely, there are some prominent inhibitors of Nrf2 activation, including CUL-3, Keap1 and GSK-3β [35], which are downregulated in our study. CUL-3 together with Keap1 forms a complex involved in Nrf2 ubiquitylation and proteasomal degradation, being a crucial regulator of its levels in the cell [50]. Under physiological conditions there is an autoregulatory loop between Nrf2 and the Keap1/CUL3 complex [51]. However, free radicals and electrophiles appear to lose this homeostatic balance. In this sense, Iso et al. [50] studied in five murine models how the amounts of Nrf2, Keap1, and Cul3 varied after exposure to the electrophilic agent diethylmaleate. Starting from a basal point in which Nrf2 was maintained at lower levels than those of Keap1 and CUL-3, the exposure to the electrophilic stimuli led to an increase of Nrf2 without affecting the levels of Keap1 and CUL-3. Hence, oxidative stress may be responsible for the enhanced levels of Nrf2 in patients with CVD, although the mechanisms involved in the homeostatic loss between this component with Keap1/CUL-3 complex remains to be elucidated. Here, we propose for future studies a possible implication of non-coding RNAs including micro RNAs (miRNAs) and long non-coding RNAs (lncRNAs), whose relevance in the regulation of oxidative stress and potential translational applications has been previously reported [52,53,54,55]. However, prior works have also described altered levels of Keap1 and CUL3 in other pregnancy complications, such as preeclampsia, suggesting its potential role in the pathophysiology of obstetric diseases [56,57]. This could entail possible pathophysiological consequences observed in the placenta of women with CVD. For instance, it seems that reduced expression of CUL-3 can be associated with matrix remodeling in the placental tissue [58], as it has been observed in the placenta of women with CVD [17]. On the other hand, low expression of Keap1 is associated with a fasted metabolic state, with noteworthy effects in the lipidome [59]. In this sense, we have previously reported that the placenta of women with CVD display a significant switch in their lipidomic profile in comparison to those without evidence of disease [60]. Further studies are warranted to deepen understanding of the pathophysiological role of these components in the placental tissue.

Finally, GSK-3β is a pivotal molecule during pregnancy involved in several biological processes in the placenta [61]. Different factors such as proper oxidative stress or PI3K/Akt hyperactivation can be responsible for the downregulation of GSK-3β in women with CVD. Additionally, previous studies have shown that reduced levels of this protein can be involved in an accelerated aging of the maternofetal structures [62,63]. On the other hand, GSK-3β may target HIF-1α to the proteasome by phosphorylation [64]. We have previously observed that the placenta tissue of women with CVD display an augmented level of HIF-1α and hypoxic stress [16]. Reduced levels of GSK-3β can be related to hypoxic events and raised HIF-1α levels observed in these patients. Despite the regulatory role of GSK-3β at multiple levels, to date there is no evidence supporting the pathophysiological role of this component in adverse outcomes of pregnancy [61]. Future efforts in the field should be directed to understand the exact mechanistic role of GSK-3β in reproductive tissues and the consequences of their alterations.

## 5. Conclusions

**Figure 5:**
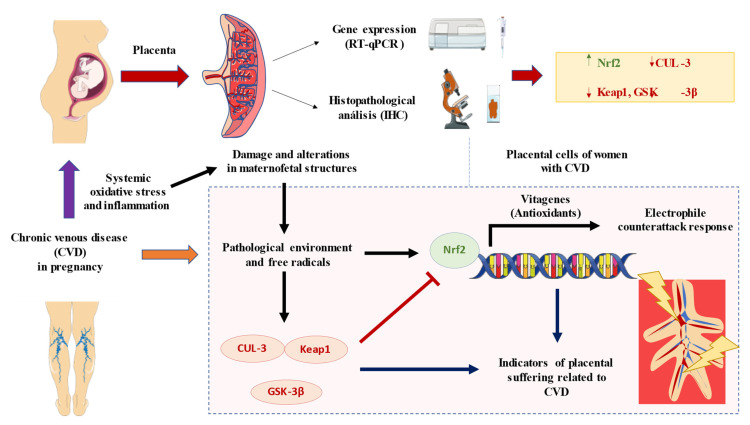
A global summary of the results obtained in this study. As shown, CVD during pregnancy is associated with systemic oxidative stress and inflammation that may have detrimental consequences for the maternofetal structures. In the present work, we have evidenced an enhanced expression of Nrf2 along with a reduced CUL-3, Keap1 and GSK-3β. Collectively, these findings suggests that the pathological environment and oxidative stress observable in the placenta of these women may lead to the dysregulation of these products, leading to the expression of several “vitagenes” involved in the electrophile counterattack response and potentially evidencing placental suffering in women with CVD.

Overall, our study has defined an aberrant gene and protein expression of Nrf2 and some of their main regulators Keap1, CUL-3 and GSK-3β in the placenta of women with CVD. As showed in Figure 5, this could be an indicator of an oxidative environment observed in this tissue, although further studies are needed to evaluate the potential consequences of these alterations.

## Figures and Tables

**Table 1:** Clinical and demographic characteristics. CVD = Chronic venous disease, HC = Healthy control.

	CVD (*n* = 62)	HC (*n* = 52)
Median age (IQR), years	33 (22–40)	34 (27–41)
Median gestational age (IQR), weeks	40.5 (39–41.5)	41 (39–42)
C-section delivery, *n* (%)	12 (19.4)	9 (17.3)
Vaginal delivery, *n* (%)	50 (80.6)	43 (82.7)
Varicose vein (CEAP), *n* (%)		
CEAP 1	37 (59.7)	0 (0)
CEAP 2	21 (33.8)	0 (0)
CEAP 3	4 (6.5)	0 (0)
Previous pregnancies, *n* (%)	33 (53.2)	19 (36.5)
Previous abortions, *n* (%)	14 (22.6)	9 (17.3)
Regular menstrual cycles, *n* (%)	50 (80.6)	42 (80.7)
Sedentary profession, *n* (%)	41 (66.1)	40 (76.9)

**Table 2:** Primer sequences used in RT-qPCR and temperature (Tm).

Gene	Sequence Fwd (5′→3′)	Sequence Rev (5′→3′)	Temp
TBP	TGCACAGGAGCCAAGAGTGAA	CACATCACAGCTCCCCACCA	60 °C
NRF2	CACGGTCCACAGCTCATCAT	GGTTGGGGTCTTCTGTGGAG	58 °C
KEAP1	TACAGCCAAGGTCCCTGAGT	CCTCAATGGACACCACCTCC	61 °C
CUL3	CGAATCTGAGCAAAGGCACG	TCCATGGTCATCGGAAAGGC	60 °C
GSK3B	GGATTCGTCAGGAACAGGACA	TTAGCATCTGACGCTGCTGT	59 °C

Gene Accession number is available in the supplementary materials.

**Table 3:** Primary and secondary antibodies used in the immunohistochemical studies performed, showing the dilutions used and the protocol specifications.

Antigen	Species	Dilution	Provider	Protocol Specifications
Nrf2	Rabbit monoclonal	1:750	Abcam (ab62352)	Glycine HCl, 30 min RT. 0.2% Hyaluronidase, 30 min 42 °C
KEAP1	Rabbit monoclonal	1:500	Abcam (ab109287)	Triton 100 × 0.1% in PBS, 10 min
CUL3	Rabbit polyclonal	1:500	Abcam (ab245410)	Glycine HCl, 30 min RT. 0.2% Hyaluronidase, 30 min 42 °C
GSK3	Rabbit monoclonal	1:250	Abcam (ab183177)	10 mM Sodium citrate pH = 6 before incubation with blocking solution
IgG (Rabbit)	Mouse	1:1000	Sigma-Aldrich (RG-96/B5283)	-

## Data Availability

The data used to support the findings of the present study are available from the corresponding author upon request.

## Supplementary Materials

The following are available online at https://www.mdpi.com/article/10.3390/antiox11112277/s1.
