# Supplementary material for: Irregular Expression of Cellular Stress Response Markers in the Placenta of Women with Chronic Venous Disease
Source: Antioxidants (Basel). 2022 Nov 17;11(11):2277. doi: 10.3390/antiox11112277 (PMC9687130; doi:10.3390/antiox11112277)
Supplement: Supplementary file 1 [file antioxidants-11-02277-s001.zip › antioxidants-1998453-supplementary.pdf]

-Homo sapiens (Human) Gene: KEAP1 (INRF2, KIAA0132, KLHL19) Q14145 KEAP1_HUMAN

-Homo sapiens (Human) Gene: NFE2L2 (NRF2) Q16236 NF2L2_HUMAN

-Homo sapiens (Human) EC:2.7.11.26 Gene: GSK3B Q6FI27 Q6FI27_HUMAN

-Homo sapiens (Human) Gene: CUL3 (KIAA0617) Q13618 CUL3_HUMAN
